# Supplementary material for: PARROT is a flexible recurrent neural network framework for analysis of large protein datasets
Source: eLife. 2021 Sep 17;10:e70576. doi: 10.7554/eLife.70576 (PMC8448528; doi:10.7554/eLife.70576)
Supplement: Supplementary file 1. — Standard error, whenever possible, is reported in parentheses. [file elife-70576-supp1.docx]

| **P.ELM** | **Metric:** | **PARROT** | **PHOSFER** | **MusiteDeep** | **PhosphoSVM** |
| --- | --- | --- | --- | --- | --- |
| **Serine**  **(S)** | **Accuracy** | **0.759 (0.003)** | 0.714 (0.003) | 0.601 (0.002) | -- |
|  | **Sensitivity** | 0.758 (0.004) | 0.843 (0.003) | **0.984 (0.001)** | 0.444 |
|  | **Specificity** | 0.761 (0.006) | 0.586 (0.006) | 0.219 (0.002) | **0.940** |
|  | **Precision** | **0.760 (0.004)** | 0.670 (0.003) | 0.557 (0.003) | -- |
|  | **F1 Score** | **0.759 (0.002)** | 0.747 (0.002) | 0.711 (0.002) | -- |
|  | **MCC** | **0.519 (0.006)** | 0.443 (0.007) | 0.314 (0.003) | 0.298 |
| **Threonine**  **(T)** | **Accuracy** | 0.729 (0.006) | 0.689 (0.005) | **0.732 (0.002)** | -- |
|  | **Sensitivity** | 0.720 (0.011) | 0.850 (0.005) | **0.864 (0.004)** | 0.373 |
|  | **Specificity** | 0.737 (0.014) | 0.529 (0.006) | 0.600 (0.006) | **0.950** |
|  | **Precision** | **0.733 (0.010**) | 0.644 (0.006) | 0.684 (0.005) | -- |
|  | **F1 Score** | 0.726 (0.007) | 0.732 (0.005) | **0.763 (0.003)** | -- |
|  | **MCC** | 0.46 (0.01) | 0.40 (0.01) | **0.481 (0.004)** | 0.251 |
| **Tyrosine**  **(Y)** | **Accuracy** | 0.630 (0.007) | 0.650 (0.008) | **0.666 (0.005)** | -- |
|  | **Sensitivity** | 0.652 (0.010) | 0.740 (0.007) | **0.835 (0.006)** | 0.419 |
|  | **Specificity** | 0.610 (0.020) | 0.561 (0.012) | 0.497 (0.009) | **0.873** |
|  | **Precision** | 0.627 (0.016) | **0.628 (0.010)** | 0.624 (0.009) | -- |
|  | **F1 Score** | 0.637 (0.007) | 0.679 (0.007) | **0.714 (0.006)** | -- |
|  | **MCC** | 0.26 (0.01) | 0.31 (0.01) | **0.353 (0.007)** | 0.209 |

**Supplemental Tables 1**: Complete table of performance metrics for phosphosite predictions on the P.ELM dataset. Standard error, whenever possible, is reported in parentheses.
