# Supplementary material for: PARROT is a flexible recurrent neural network framework for analysis of large protein datasets
Source: eLife. 2021 Sep 17;10:e70576. doi: 10.7554/eLife.70576 (PMC8448528; doi:10.7554/eLife.70576)
Supplement: Supplementary file 2. — Standard error, whenever possible, is reported in parentheses. [file elife-70576-supp2.docx]

| **PPA** | **Metric:** | **PARROT** | **PHOSFER** | **MusiteDeep** | **PhosphoSVM** |
| --- | --- | --- | --- | --- | --- |
| **Serine**  **(S)** | **Accuracy** | 0.699 | **0.695** | 0.582 | -- |
|  | **Sensitivity** | 0.625 | 0.784 | **0.946** | 0.340 |
|  | **Specificity** | 0.773 | 0.606 | 0.217 | **0.959** |
|  | **Precision** | **0.734** | 0.666 | 0.547 | -- |
|  | **F1 Score** | 0.675 | **0.720** | 0.693 | -- |
|  | **MCC** | **0.402** | 0.397 | 0.239 | 0.237 |
| **Threonine**  **(T)** | **Accuracy** | 0.598 | **0.617** | 0.597 | -- |
|  | **Sensitivity** | 0.399 | **0.618** | 0.564 | 0.218 |
|  | **Specificity** | 0.797 | 0.616 | 0.631 | **0.934** |
|  | **Precision** | **0.663** | 0.617 | 0.604 | -- |
|  | **F1 Score** | 0.498 | **0.617** | 0.583 | -- |
|  | **MCC** | 0.21 | **0.23** | 0.195 | 0.116 |
| **Tyrosine**  **(Y)** | **Accuracy** | 0.560 | **0.564** | 0.545 | -- |
|  | **Sensitivity** | 0.455 | 0.517 | **0.574** | 0.286 |
|  | **Specificity** | 0.664 | 0.611 | 0.516 | **0.844** |
|  | **Precision** | **0.575** | 0.571 | 0.542 | -- |
|  | **F1 Score** | 0.508 | 0.543 | **0.558** | -- |
|  | **MCC** | 0.122 | **0.129** | 0.090 | 0.084 |

**Supplemental Tables 2**: Complete table of performance metrics for phosphosite predictions on the PPA datasets. Standard error, whenever possible, is reported in parentheses.
