# Supplementary material for: PARROT is a flexible recurrent neural network framework for analysis of large protein datasets
Source: eLife. 2021 Sep 17;10:e70576. doi: 10.7554/eLife.70576 (PMC8448528; doi:10.7554/eLife.70576)
Supplement: Supplementary file 3. — Datasets were created by assigning random values in [–5, 5] to randomly generated protein sequences ~25–35 residues in length. Networks were trained using a NVIDIA TU116 GPU. Three replicate PARROT networks were trained for each specified set of hyperparameters and dataset. [file elife-70576-supp3.docx]

| **Layers** | **Vector size** | **5K Sequences** | **10K Sequences** | **50K Sequences** | **100K Sequences** |
| --- | --- | --- | --- | --- | --- |
| 1 | 2 | 16 s | 29 s | 133 s | 262 s |
| 1 | 10 | 15 s | 30 s | 134 s | 268 s |
| 1 | 25 | 16 s | 29 s | 133 s | 271 s |
| 3 | 2 | 29 s | 59 s | 275 s | 559 s |
| 3 | 10 | 30 s | 59 s | 280 s | 557 s |
| 3 | 25 | 31 s | 59 s | 279 s | 564 s |
| 5 | 2 | 42 s | 85 s | 414 s | 844 s |
| 5 | 10 | 44 s | 86 s | 416 s | 844 s |
| 5 | 25 | 44 s | 86 s | 417 s | 846 s |

**Supplemental Table 3**: Average PARROT network training times on different sizes of datasets and with variable hyperparameters. Datasets were created by assigning random values in [-5, 5] to randomly generated protein sequences ~25-35 residues in length. Networks were trained using a NVIDIA TU116 GPU. Three replicate PARROT networks were trained for each specified set of hyperparameters and dataset.
